# Designing Transmission Strategies for Enhancing Communications in Medical IoT Using Markov Decision Process

**DOI:** 10.3390/s18124450

**Published:** 2018-12-15

**Authors:** Moumita Roy, Chandreyee Chowdhury, Nauman Aslam

**Affiliations:** 1 Computer and Information Sciences, Jadavpur University, Kolkata 700032, India; moumita1055@gmail.com (M.R.); chandreyee.chowdhury@gmail.com (C.C.); 2 Computer Information Sciences, Northumbria University, Newcastle Upon Type NE1 8ST, UK

**Keywords:** medical IoT, WBAN, energy efficient, Markov Decision Process, routing, specific absorption rate, transmission strategy

## Abstract

The introduction of medical Internet of Things (IoT) for biomedical applications has brought about the era of proactive healthcare. Such advanced medical supervision lies on the foundation of a network of energy-constrained wearable or implantable sensors (or things). These miniaturized battery-powered biosensor nodes are placed in, on, or around the human body to measure vital signals to be reported to the sink. This network configuration deployed on a human body is known as the Wireless Body Area Network (WBAN). Strategies are required to restrict energy expenditure of the nodes without degrading performance of WBAN to make medical IoT a green (energy-efficient) and effective paradigm. Direct communication from a node to sink in WBAN may often lead to rapid energy depletion of nodes as well as growing thermal effects on the human body. Hence, multi-hop communication from sources to sink in WBAN is often preferred instead of direct communication with high transmission power. Existing research focuses on designing multi-hop protocols addressing the issues in WBAN routing. However, the ideal conditions for multi-hop routing in preference to single-hop direct delivery is rarely investigated. Accordingly, in this paper an optimal transmission policy for WBAN is developed using Markov Decision Process (MDP) subject to various input conditions such as battery level, event occurrence, packet transmission rate and link quality. Thereafter, a multi-hop routing protocol is designed where routing decisions are made following a pre-computed strategy. The algorithm is simulated, and performance is compared with existing multi-hop protocol for WBAN to demonstrate the viability of the proposed scheme.

## 1. Introduction

**Figure 1:**
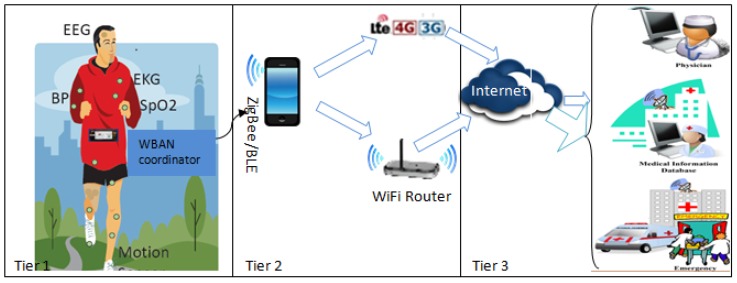
Three-tier architecture of WBAN.

Medical Internet of Things (IoT) [1] enables a collection of medical devices and applications to be connected through the Internet, and has revolutionized the conventional concept of healthcare. Wearable applications of IoT in the medical field has spawned the era of smart healthcare [2,3], which enables constant medical supervision under free living conditions and thus upgrades the existing medical infrastructure. A three-tier architecture [2] (as shown in Figure 1)—based proactive healthcare approach could enhance the quality of living in different ways [3] by providing continuous medical assistance at a reasonable cost. Such a health-monitoring system subject to an energy constraint network, namely Wireless Body Area Network (WBAN) [2], a network of small-size, ultra-low-power, wearable or implantable biosensor nodes powered by batteries placed in, on, or around the human body to measure vital physiological parameters together with a WBAN coordinator (that could be a smart handheld device as well), acts as a sink to store and process health data locally. Tier 1 focuses on network formation, whereas in tier 2, the sink communicates via WLAN or GPRS technology to a remote medical server residing at tier 3 for analysis of health data by medical personnel and to provide necessary actions.

In tier 1 of WBAN, different applications could use a suitable physical and MAC layer as defined by IEEE standards. These include IEEE 802.15.4 (Zigbee), IEEE 802.15.1 (Bluetooth) and IEEE 802.15.6 (mainly used for implantable nodes). To prolong the lifetime [4] of the network to achieve long-lasting benefits of WBAN applications [2,5] energy-efficient network layer [6,7] is essential. The low-power-consumption (typically, power output rating lower than 0 dBm) feature of BAN transceivers limits transmission distances to around 2 m, depending on power output and environmental characteristics [8]. Hence, relay nodes are often placed in the network to acquire the benefits of short-range multi-hop communication [4,7,9] with low transmission power over long-distance high-power direct data delivery. However, the latest version of the IEEE standard proposed for WBAN in February 2012 suggests two-hop communication [2]. Although a proprietary system could use more than two hops, in such cases, interoperability could be a significant challenge.

Research works reported in [2,10] identified that data transmission is the prime source of energy expenditure within WBAN and hence strategies should be made to obtain a balance between reliable data delivery with minimal energy depletion. Previous research works reported in [11,12,13,14] addressed the key factors related to the system state such as energy level of nodes, event occurrence, energy-harvesting capacity, data importance etc. to carry out analytical formulation of transmission strategies. Mathematical models, particularly Markov Decision Process (MDP) [15], is found to be exploited in state-of-the-art works reported in [12,13] to predict the optimal sequence of actions to be followed to achieve the desired goal. In addition, the type of nodes used in WBAN are either implanted or wearable and thus data communication takes place through tissue and/or air medium accordingly [3]. Hence, channel conditions impose an impact on reliable data delivery. Low power transmission in adverse channel conditions could result in data loss. Besides, electromagnetic radiation absorbed by human tissue (measured in terms of Specific Absorption Rate (SAR), [2]) generated due to network activities of bio sensor nodes may result in several health hazards [16] if it reaches beyond the regulatory limit [2]. However, the heat generation is proportional to the energy depletion rate of the participatory nodes in the network which in turn subjects to the effective transmission power of data communication. Thus, obtaining optimal transmission power for intra-BAN communication plays a key role in effective network design. However, this analytical approach towards designing optimal transmission strategy requires post-deployment feedback as well to justify its effectiveness. Another trend is observed in literature [17,18] to address these pivotal issues related to data transmission within WBAN during routing data towards the sink. Here, optimal transmission power is chosen adaptively at runtime as part of the routing decisions. Hence, in this case the complex mathematical analysis to obtain optimal transmission power could over-burden such resource-constrained network.

In this work, a transmission strategy for multi-hop intra-BAN communication has been formulated offline prior to network deployment based on the following input conditions: energy level of nodes, event occurrence depicted in terms of data packet generated by nodes to deliver to sink, packet transmission rate which reflects the effect of heat generation due to network activities and link quality. The designed policies are incorporated into the nodes and thereafter a multi-hop routing protocol has been devised where routing decision follows the pre-computed strategy. In this work the contributions are as follows.

- An MDP formulation to develop the transmission strategy for multi-hop communication within WBAN which not only focuses on obtaining optimal transmission power subject to the input conditions but also reflects the necessity of multi-hop data transmission as well.
- A routing algorithm is designed based on the effective transmission strategies obtained by solving MDP formulation prior to deployment. These transmission strategies correspond to the system states that the network may undergo after deployment in terms of energy level, event occurrence, packet transmission rate and link quality. The nodes route data following a simple but effective routing algorithm and make a decision to transmit via multi-hop or single-hop based on suitable transmission power.
- The effectiveness of the designed solutions is verified with extensive simulations, and performance is evaluated with respect to the existing multi-hop protocol for WBAN.

The paper is organized as follows. Section 2 provides an overview of the state-of-the-art works reported in literature followed by a discussion of MDP in Section 3. Section 4 presents the proposed work in detail along with the system model and MDP formulations. Section 5 illustrates the experimental particulars including the simulation setup and analysis of experimental results. Finally, concluding remarks are presented in Section 6.

## 2. Related Work

A vast literature could be found on energy efficiency and lifetime improvement of WSN. Although WSNs significantly differ from WBANs in many aspects including size, scope, application, coverage etc., understanding of research efforts in traditional WSNs is important to gain deeper insight and full context. Hence, the first part of this section discusses energy efficiency in traditional WSNs for completeness. Grouping nodes to form clusters is one of the well-known mechanisms for energy-efficient communications in WSN. A clustering-based protocol LEACH (Low-Energy Adaptive Clustering Hierarchy) was proposed by Heinzelman et al. [19] that exploited randomized rotation of cluster heads to evenly distribute the energy load among the sensors in the network. In-LEACH scalability and robustness for dynamic networks are addressed by localized coordination, and data fusion is found to be incorporated into the routing protocol to reduce the amount of information to be transmitted to the base station. Another distributed clustering approach was proposed by Younis et al. [20] for long-lived sensor networks. This approach does not make any assumptions for factors like the presence of infrastructure, or the availability of multiple power levels in sensor nodes. Instead, a Hybrid Energy-Efficient Distributed clustering (HEED) was presented by the authors. In this protocol cluster heads are selected periodically based on the residual energy of a node and node proximity to its neighbors or node degree. The advantages and objectives of clustering for WSN are analyzed by Liu [21] along with a comparative study of these protocols.

There are works reported in literature that focus on designing transmission strategies for WSN to achieve energy efficiency. In [11] a generic mathematical framework was proposed to characterize the policies for single-hop transmission over a replenishable sensor network. Here different modes of energy renewal were presented with Markov Chain Process and accordingly, optimal transmission policy was derived for sensors with different energy budgets. The energy status and the reward for successfully transmitting a message were given as input to the formulations to maximize the average reward rate and justify the existence of optimal threshold as well. In [22] optimal selective forwarding policies was proposed to save energy and extend the lifetime of WSN based on the available energy resources of the nodes, the energy cost of retransmitting a message or the importance of the message. Forwarding schemes included three different scenarios: first, when sensors maximized the importance of their own transmitted message; second, when the sensors maximized the importance of messages that were successfully retransmitted by at least one of its neighbors; and finally, when sensors maximized the importance of messages that successfully arrived at the sink. Performance was measured in terms of gain of selective forwarding policies under exponential importance distribution when energy costs were stochastic. Thus, the current energy level of nodes is found to be the key element in modeling transmission strategy in the works reported in [11,12,13]. However, data importance is obtained as another input parameter for optimal transmission strategy formulation in the works reported in [13,22,23]. In [13] policies were developed whether to transmit the data packet or not, based on the current energy level and data importance, to maximize the long-term average transmitted data importance. Whereas, the work in [24] aimed at maximizing the long-term average transmission rate considering energy-harvesting device with packet data queue.

However, the architecture, deployment area, and operating conditions of WSNs differ from WBANs. WBANs are deployed on a human body thus having limited coverage area but sensitive to transmission energy (as radiation may cause tissue damage). On the contrary, in WSNs, hundreds of sensor nodes cover large areas such as an agricultural field or a city and use multi-hop communications. Thus, clustering can be a useful solution for WSNs but for WBANs with typically 6–20 nodes (standard for typical medical network [2]), it can add unwanted overheads. The body sensors require mostly single-hop or two-hop data transmission. Not only node lifetime, but also the rate of energy depletion due to computational and transmission power is important for WBAN as these factors directly correspond to SAR.

Existing research works on development of transmission strategies are listed in Table 1 according to time line. The works in [12,14,24] primarily focused on analytical development of transmission strategies for intra-BAN data communications. In [17,18], authors reported the development of network layer protocols which incorporate the adaptive selection of transmission power as a component of the routing process. An energy-efficient fuzzy routing protocol was proposed in [17] which attempted to prolong the lifetime of the network by taking the optimal route to a destination based on energy level, traffic load and link usage. The following steps are found to be executed by the protocol proposed in [17]. At the beginning, nodes access their own battery level. Next, transmission power is adjusted depending on distance and subsequently, routing queries are generated to transfer data. Thereafter, a single neighbor’s information is obtained, and routing path is calculated. Finally, a fuzzy inference system is invoked to take the decision about the next hop. Here the node with low energy is avoided to act as the next hop for the data packets to be delivered to the sink. The transmission power is adjusted in the work according to the distance of the receiver node. However, adaptive power control and routing in multi-hop WBANs are considered in [18] to develop a low overhead energy-efficient routing scheme based on Collection Tree Protocol (CTP). However, these protocols attempted to obtain optimal transmission power during network activities after deployment of body-sensor nodes which could impose an additional overhead to the resource-constrained system. Nevertheless, the use of mathematical models in developing transmission strategies have been a common practice over the years. The works reported in [11,12,13,14,22,24] are found to exploit MDP [15] to formulate transmission policies whereas in [25] Monte Carlo Simulation is used to design energy-efficient adaptive transmission power control scheme. Network-coded transmission policies are presented in [26] to reduce the number of transmissions in simple multi-hop networks. However, the existing literature mostly uses MDP to design transmission policy as this is found to be the convenient mathematical framework for planning under uncertainty. Hence, Table 1 refers to the existing works formulated with MDP only.

In [27], authors reported a system level energy consumption model associated with transmission distance and transmission data rate over on body communication link. Then, they derived a threshold distance for energy saving in WBAN based on the analysis of tradeoff between circuit energy and transmission energy. According to the work in [27], for a distance less than or equal to threshold distance, circuit energy is comparable to the transmission energy consumption and as such total energy expenditure could be restricted by optimizing the transmission data rate.

A two-hop communication system with energy-harvesting nodes was presented in [29] where both source and relay were able to harvest energy from environment during communication. The works in [13,14] also aimed at harvesting energy from ambient resources apart from conventional objectives such as energy efficiency, reliability, throughput maximization etc. A comprehensive taxonomy of the various energy-harvesting sources in WSN was presented by the authors in [30]. In [12,31], authors included energy-harvesting process as an input criterion to develop optimal transmission policy as well. In [12], authors devised a transmission strategy by taking into account energy level of nodes, event generation, battery recharge and packet error probability. Here two transmission modes are assumed to be available for the sensors allowing tradeoff between energy consumption and packet error probability. Decision policies are formulated here to obtain the transmission mode to be used at a given instant of time to maximize the quality of coverage. The problem was structured exploiting MDP framework and an upper bound in the performance of arbitrary policies was figured out. However, in [31], authors considered energy replenishment process and battery capacity to find optimal transmission policies for rechargeable nodes. Here optimal solutions have been identified for two related problems; first the transmission policy that maximizes the short-term throughput in terms of the amount of data transmitted in a finite time horizon is obtained and next minimization of the transmission completion time for a given amount of data is addressed accordingly. In this work, a model with discrete packets of energy arrival has been considered for battery replenishment. However, the recharging or replacement of batteries of body-sensor nodes is not always feasible in the case of WBAN particularly in case of implanted nodes. A cross layer design was proposed in [7] to address the pivotal issues of WBAN communication such as transmission reliability, energy efficiency, lifetime grounded on transmission power control, relay decision and packet selection within WBAN. The work adopted cross layer design involving physical, MAC as well as network layer. The work is found to focus on choosing optimal transmission power by maximizing energy efficiency over a single link and after that optimal relay is decided through the tradeoff between maximization of energy and minimization of energy consumption speed. Next, remaining energy of leaf nodes of WBAN topology is exploited to enhance transmission reliability without any loss of lifetime. At the end, the optimized packet size has been selected for optimizing energy efficiency.

Thus, the existing works opt for developing transmission strategies for energy-constrained networks (i.e., WSN and WBAN) particularly focusing on finding optimal transmission power subject to different input conditions such as current energy level, event generation, data importance etc. Thus, prime issues related to communication (such as energy efficiency, reliability, throughput etc.) along with energy harvesting (to cope up with scarcity of energy) can be addressed. Initially, research was carried out focusing on one or two input conditions, but more dimensions were gradually added for better analysis. In addition, previously most of the approaches were designed to be used for single-hop data transmission but later, the trend moved towards multi-hop scenario. Still there is room for further exploration of modeling multi-hop strategy. Besides, the transmission power has severe impact on heat generation in WBAN which is hardly investigated in state-of-the-art literature. The transmission policies framed for WSN cannot be applied directly in WBAN due to its inherent challenges as well. Furthermore, use of mathematical model with intricate numerical formulations to predict optimal strategy could intensify the complexity of resource-constrained network. Herewith, in this paper energy-efficient multi-hop transmission strategy following MDP is proposed for intra-BAN communication which predicts optimal policy prior to deployment of the network. The outcome is incorporated into the nodes to get reflected in routing decisions during post-deployment phase.

## 3. Markov Decision Process

**Figure 2:**
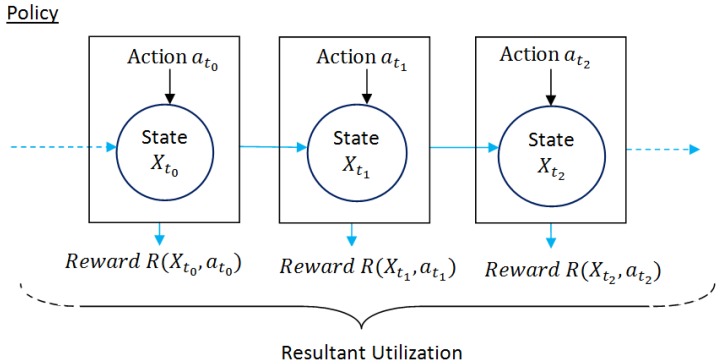
MDP Process Model where $t_{i}$ denotes *i*-th time instant.

MDP [15] is described as a discrete-time-state transition stochastic process which gives a mathematical framework for making any rational decision when the outcome is partly random and partly regulated by the decision makers [27]. It is convenient to use MDP formulations to line up a strategy under uncertainty. Here decisions are optimized in either of the following way, i.e., minimization of the expected cost to meet the objective or maximization of the expected reward. MDP process model is presented in Figure 2.

**Figure 3:**
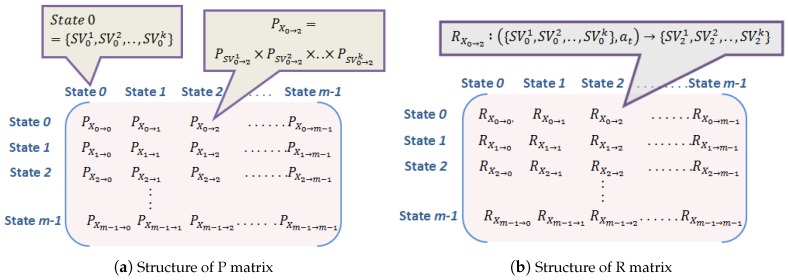
Structure of State transition matrix (P) and corresponding reward matrix (R) for m system states subject to an action (*a_t_*).

MDP is expressed by five-tuple: $\left(X_{t},A_{t},P,R,\gamma^{\prime }\right)$. Here $X_{t}$ denotes system state at any time instant *t* and $A_{t}$ represents the finite set of actions ($a_{t}$) where the corresponding action $a_{t}$ if performed at $X_{t}$ drives the system state $X_{t}$ to one of the probable next states $X_{t+1}$ as depicted in Figure 2. The state transition probabilities from each system state $X_{t}$ to next possible state $X_{t+1}$ depending on the action $a_{t}$ performed are recorded in matrix P and the corresponding reward generated at each state are documented in matrix R. The structure of P matrix and R matrix are presented in Figure 3a,b respectively for m number of system states subject to an action $a_{t}$. Both matrices are represented in m×m dimensions indicating all possible state changes where each row heading and column heading, i.e., $State\;i\;\exists_{i=0}^{m-1}$ indicate present system state and next possible system state, respectively. For instance, the element of P matrix $P_{X_{0\to 2}}$ represents state transition probability from state 0 to state 2 and accordingly the element $R_{X_{0\to 2}}$ of R matrix denotes the corresponding reward generated due to state change from state 0 to state 2 depending on the action $a_{t}$. However, each system state could be represented as a combination of k state variables

(1) $$X=\{SV^{1},SV^{2},\ldots ,SV^{k}\}$$

Each state variable could have different range of values, say $m_{1}$, $m_{2}$, …, $m_{k}$ then the product of these values gives the order of these matrices, i.e., $m=m_{1}\times m_{2}\times \ldots \times m_{k}$. For simplicity, let State 0 and State 2 are represented as $\left\{SV_{0}^{1},SV_{0}^{2},\ldots ,SV_{0}^{k}\right\}$ and $\left\{SV_{2}^{1},SV_{2}^{2},\ldots ,SV_{2}^{k}\right\}$ respectively in Figure 3a,b. In such case, the element of P matrix $P_{X_{0\to 2}}$ is given by the product of state transition probabilities of each state variables from State 0 to State 2, i.e., $P_{X_{0\to 2}}=P_{{SV^{1}}_{0\to 2}}\times P_{{SV^{2}}_{0\to 2}}\times \ldots \times P_{{SV^{k}}_{0\to 2}}$. Accordingly, the reward $R_{X_{0\to 2}}$ in R matrix gives the reward value which results due to performing action $a_{t}$ at $State0(\left\{SV_{0}^{1},SV_{0}^{2},\ldots ,SV_{0}^{k}\right\})$ which drives the system to $State2(\left\{SV_{2}^{1},SV_{2}^{2},\ldots ,SV_{2}^{k}\right\})$. Hence, a pair of P matrix and R matrix are required for each action.

The series of rewards obtained due to performing sequence of actions at each predicted state starting from the current state yield the utility value. Obtained rewards are simply put in to quantify additive utility, i.e.,

(2) $$U_{add}\left(\left[r_{0},r_{1},r_{2}\ldots \right]\right)=r_{0}+r_{1}+r_{2}+\ldots$$

whereas discounted utility is measured using a discount factor ($\gamma^{\prime }<1$) where sooner rewards have more impact than later rewards.

(3) $$U_{dis}\left(\left[r_{0},r_{1},r_{2}\ldots \right],\gamma^{\prime }\right)=r_{0}+\gamma^{\prime }r_{1}+{\gamma^{\prime }}^{2}r_{2}+\ldots$$

Discounted utility is particularly suitable for convergence of optimization algorithms where the sequence of actions is predicted to intensify the expected discounted utility. Optimal MDP policy is designed by value iteration process that is repeated for all states *s*. An arbitrary value $V_{0}$ assigned at each state. $V_{n+1}\left(s\right)$ is enumerated exploiting Bellman backup at *s* [15] such that the iteration process continues until ϵ-convergence is meet, i.e., $max_{s}\left|V_{n+1}\left(s\right)-V_{n}\left(s\right)\right|<\epsilon$. State transitions matrix P and reward matrix R together with discount factor ($\gamma^{\prime }<1$) are fed as input to the value iteration process to acquire the discounted utility value along with the number of iterations. Next, finite horizon method is employed which takes the number of iterations obtained from value iterations process along with other inputs as in value iteration process, (i.e., $P,R,\gamma^{\prime }$) and results in non-stationary policies (π). Thus, MDP is pertinent to foresee the optimal course of actions initiating from the present state to accomplish utmost benefit.

## 4. Our Work

In this paper, the entire work is carried out in two phases. Phase I presents the work done in pre-deployment phase. Markov Decision Process [15]-based mathematical formulation is presented in this phase to design multi-hop transmission strategy to predict optimal sequence of actions to be performed subject to the input conditions, i.e., energy level of nodes, event occurrence, packet transmission rate and link quality. This is followed by Phase II where a multi-hop routing protocol has been devised which reflects the obtained transmission policies of Phase I in the routing decisions to address the prime issues of WBAN communications.

### 4.1. System Model

A network is built with *n* bio sensor nodes, and single sink node which acts as network coordinator and accumulates the data from the sensor nodes to communicate to the remote medical server. Two nodes are assumed to be implanted inside human body near heart and right knee and rest (n-2) wearable nodes are assumed to be placed on human body (discussed in Section 5.2). The nodes in the network transmit data with transmission power $P_{tx}$ governed by the proposed transmission strategy following MDP [15] that is elaborated in the following subsection. Intra-BAN communications are carried out via electromagnetic radio frequency (RF) wave. The radiation absorbed by human tissue is quantified in terms of SAR (Watts/Kg). SAR is evaluated for each node in the network due to performing network activities. SAR assessment is based on the effective distance ‘*d*’ from each node location to the reference point in the human tissue. If the reference point is located at the near field region with respect to a node, SAR is evaluated as follows [32]

(4) $$SAR=\frac{\sigma }{\rho }\frac{\mu \omega }{\sqrt{\sigma^{2}+\epsilon_{1}^{2}\omega^{2}}}{\left(\frac{Ilsin\theta }{4\pi }e^{-\alpha d}\left(\frac{1}{d^{2}}+\frac{|\gamma_{1}|}{d}\right)\right)}^{2}$$

Here $\sigma ,\epsilon_{1},\mu$ represent conductivity (S/m), permittivity (F/m) and permeability (H/m) of the medium, respectively. $\gamma_{1}$ is the complex propagation constant. α is the attenuation constant and ρ is the density of the medium. *l* is the dipole length and current *I* is uniform and varies sinusoidally with time. However, if the location of the reference point is at far field region with respect to a node, SAR is formulated as follows [32]

(5) $$SAR=\frac{\sigma }{\rho }\left(|\eta |\right|\gamma_{1}{\left|\frac{Idlsin\theta }{4\pi d}e^{-\alpha d}\right)}^{2}$$

where η is complex intrinsic impedance defined as $\eta =\frac{\gamma_{1}}{\alpha +j\omega \epsilon_{1}}$.

A discrete-time model has been taken into account where time is slotted in intervals of unit length. Each node can generate and transmit a single data packet per time slot. Data transmission between source and sink is described as event occurrence that is defined with correlated, two-state process.

The remaining energy of each node is classified into several levels $L_{t}$ depending on predefined range (to be obtained empirically) such that at any time slot t remaining energy of each node belongs to one of the defined energy levels $L_{t}\epsilon \left\{0,1,2,3,4\ldots N\right\}$.

If an event is generated in the present slot, probability of generation of another event (respectively, no event) in the next slot is given by $p_{on}$ (respectively, $1-p_{on}$) where $0.5<p_{on}<1$ [12]. If no event is generated in the present slot, an event is generated (respectively, not generated) in the next slot with probability $1-p_{off}$ (respectively, $p_{off}$) where $0.5<p_{off}<1$ [12]. However, in both cases the probability of event generation and no event at present time slot are two complementary as well as equally likely outcomes. Hence, the value of $p_{on}$ and $p_{off}$ lies between 0.5 to 1. During each time slot Packet Transmission Rate (PR) is compared with a limiting value, i.e., $PR_{th}$. If the PR reaches beyond threshold $PR_{th}$ in the current slot, it will be greater than (respectively, less than) $PR_{th}$ in the subsequent slot with probability $pr_{on}$ (respectively, $1-pr_{on}$) where $0.5\leq pr_{on}<1$. However, if the PR is less than the threshold $PR_{th}$ in the present slot, it will remain less than (respectively, greater than) $PR_{th}$ in the following slot with probability $pr_{off}$ (respectively, $1-pr_{off}$) where $0.5\leq pr_{off}<1$.

Link quality (LQ) is measured at each time slot to estimate channel conditions as well and if it is found high in the present slot, i.e., greater than predefined threshold $LQ_{th}$, it will remain high in the following slot with probability $lq_{on}$ (respectively, $1-lq_{on}$) where $0.5\leq lq_{on}<1$. Whereas, if LQ is estimated as low as $\left(LQ\;<\;LQ_{th}\right)$ in the current slot, then it will be in such condition in the next slot with probability $lq_{off}$ (respectively, $1-lq_{off}$). Such a two-state model can effectively describe the scenario for many WBAN applications related to healthcare as mentioned in [12].

During each time slot t a node performs action $a_{t}$ s.t. $a_{t}\epsilon \left\{0,1,2,3,\ldots K\right\}$ which is described in terms of performing data transmission with varying transmission power $P_{tx}$.

### 4.2. Phase I (Pre-Deployment Phase)

#### Markov Decision Process Formulation

The optimal transmission power $P_{tx}$ in each slot is determined following MDP [15] subject to four prime aspects, i.e., current battery level of the node, event occurrence, data PR and LQ. Consequently, at any time slot t the system state is represented by

(6) $$X_{t}=\left(L_{t},E_{t},PR_{t},LQ_{t}\right)$$

where $L_{t}\epsilon \left\{0,1,2,3,4,\ldots ,N\right\}$ denotes the energy level of sensor node at time slot t. $E_{t}\epsilon \left\{1,0\right\}$ represents an event to be reported in other words whether a node has packet to transmit at time t. For instance, when a data packet is received from upper layer ready for transmission, $E_{t}$ is 1. It is assumed that at-most one data packet is generated by each node per time slot. $PR_{t}\epsilon \left\{1,0\right\}$ indicates if data packet transmission rate is low or high; high packet transmission rate intensifies energy depletion rate which in turn increase SAR. $PR_{t}$ is 1 if $PR_{t}\;\geq \;PR_{th}$ at present slot and 0 otherwise. Here, $PR_{th}$ is directly related to SAR threshold of a node specified by an application. Finally, $LQ_{t}\epsilon \left\{1,0\right\}$ denotes link quality where $LQ_{t}$ is 1 for stable channel conditions at present slot such that

$$\frac{\phi_{i}}{\left(\delta_{0}+\delta_{i}\right)}>\frac{\phi_{i+1}}{\left(\delta_{0}+\delta_{i+1}\right)}\;s.t.\;\;i\epsilon \left\{1,2,\ldots ,N\right\}$$

and 0 otherwise. Here $\delta_{0}$ quantifies the energy depleted to run the circuitry and $\delta_{i}$ for 0<i≤N gives the amount of required energy (in addition to $\delta_{0}$) for data transmission with transmission power $P_{tx}$ and $\phi_{i}$ represents corresponding packet success rate.

Energy level $L_{t}$ changes according to the action $a_{t}$ taken at time slot t, where $a_{t}\epsilon \left\{0,1,2,3,\ldots ,K\right\}$.

(7) $$\begin{array}{lll} L_{t} & = & 0\;\mathrm{if}\;E_{rem}\left(t\right)=\delta_{0}\\ & = & i\;\mathrm{if}\;E_{rem}\left(t\right)=\delta_{0}+\delta_{i}\;\mathrm{where}\;1\leq i\leq N \end{array}$$

Following MDP, an action $a_{t}$ is performed at each state $X_{t}$ that takes the system to the next state $X_{t+1}$ while resulting in a reward $R(X_{t},a_{t})$ as shown in Figure 2. Solving the MDP formulation gives us the actions to be performed in each iteration that results in maximum reward. It is to be noted that calculation of both next state and reward only depend on the current state and the action taken at that state. Thus, at any time slot *t* reward (*R*) is quantified as the probabilities of successful data delivery subject to the input conditions, i.e., possible combination of energy level, event occurrence, PR and channel conditions.

(8) $$R(X_{t},a_{t})=\{\begin{array}{ll} (1-p_{off})\times pr_{off}\times (1-lq_{off}), & \mathrm{if}\;E_{t-1}=0\;\&PR_{t-1}=0\;\&\\ & LQ_{t-1}=0\;\&L_{t}>\delta_{0}+\delta_{i}\\ (1-p_{off})\times pr_{off}\times lq_{on}, & \mathrm{if}\;E_{t-1}=0\;\&PR_{t-1}=0\;\&\\ & LQ_{t-1}=1\;\&L_{t}>\delta_{0}+\delta_{i}\\ (1-p_{off})\times (1-pr_{on})\times (1-lq_{off}), & \mathrm{if}\;E_{t-1}=0\;\&PR_{t-1}=1\;\&\\ & LQ_{t-1}=0\;\&L_{t}>\delta_{0}+\delta_{i}\\ (1-p_{off})\times (1-pr_{on})\times lq_{on}, & \mathrm{if}\;E_{t-1}=0\;\&PR_{t-1}=1\;\&\\ & LQ_{t-1}=1\;\&L_{t}>\delta_{0}+\delta_{i}\\ p_{on}\times pr_{off}\times (1-lq_{off}), & \mathrm{if}\;E_{t-1}=1\;\&PR_{t-1}=0\;\&\\ & LQ_{t-1}=0\;\&L_{t}>\delta_{0}+\delta_{i}\\ p_{on}\times pr_{off}\times lq_{on}, & \mathrm{if}\;E_{t-1}=1\;\&PR_{t-1}=0\;\&\\ & LQ_{t-1}=1\;\&L_{t}>\delta_{0}+\delta_{i}\\ p_{on}\times (1-p_{on})\times (1-lq_{off}), & \mathrm{if}\;E_{t-1}=1\;\&PR_{t-1}=1\;\&\\ & LQ_{t-1}=0\;\&L_{t}>\delta_{0}+\delta_{i}\\ p_{on}\times (1-pr_{on})\times lq_{on}, & \mathrm{if}\;E_{t-1}=1\;\&PR_{t-1}=1\;\&\\ & LQ_{t-1}=1\;\&L_{t}>\delta_{0}+\delta_{i}\\ 0, & \mathrm{Otherwise} \end{array}$$

The system state in next time slot (t+1) is denoted by

(9) $$X_{t+1}=\left(L_{t+1},E_{t+1},PR_{t+1},LQ_{t+1}\right)$$

Energy level in the next slot is given by

(10) $$L_{t+1}=L_{t}-l_{t}\;s.t.\;L_{t+1}\leq L_{t}$$

where $l_{t}$ is the amount of energy consumed due to data transmission.

(11) $$\begin{array}{ll} l_{t}= & i\;w.p.\;\left[E_{t}\times p_{on}+\left(1-E_{t}\right)\times \left(1-p_{off}\right)\right]I_{i-1}\left(a_{t}\right)\\ & \mathrm{if}\;E_{rem}\left(t\right)>\delta_{0}+\delta_{i}\;\;\&\;1\leq i\leq N \end{array}$$

here $I_{y}\left(a_{t}\right)$ represents the indicator function that equals one only when value of $a_{t}$ equals the subscript *y* and zero otherwise. In the equation, “w.p.” stands for with probability. Event generation in the next time slot (*t* + 1) is predicted as

(12) $$\begin{array}{lll} E_{t+1} & = & 1\;w.p.\;\;[E_{t}\times p_{on}+\left(1-E_{t}\right)\times \left(1-p_{off}\right)]\\ & = & 0\;Otherwise \end{array}$$

Accordingly, whether packet transmission rate PR exceeds $PR_{th}$ in the next time slot (*t* + 1) is evaluated as

(13) $$\begin{array}{lll} PR_{t+1} & = & 1\;w.p.\;[PR_{t}\times pr_{on}+\left(1-PR_{t}\right)\times \left(1-pr_{off}\right)]\\ & = & 0\;Otherwise \end{array}$$

Likewise, link quality LQ in the next time slot is predicted as

(14) $$\begin{array}{lll} LQ_{t+1} & = & 1\;w.p.\;[LQ_{t}\times lq_{on}+\left(1-LQ_{t}\right)\times \left(1-lq_{off}\right)]\\ & = & 0\;Otherwise \end{array}$$

At any time slot *t* with the current state $X_{t}$ the transition probability for $X_{t+1}$ is found using Equations (11)–(14). A matrix P is constructed to record the state transition probabilities from each of the current state $X_{t}$ to its corresponding next state $X_{t+1}$ depending on the working condition of a node in terms of $L_{t}$, $E_{t}$, $PR_{t}$ and $LQ_{t}$. At each system state $X_{t}$, some action $a_{t}$ is performed which results in certain reward (as illustrated in Equation (9)) and accordingly a reward matrix R is formed with all possible rewards corresponding to a system state $X_{t}$ for each action $a_{t}$. This MDP formulation is solved using value iteration technique [15]. For any stationary policy $\pi =(\pi_{0},\pi_{1},\ldots )$, the state value function at a state x∈X satisfies the Bellman equation [15],

(15) $$V^{\pi }\left(x\right)=R\left(x,\pi \left(x\right)\right)+\gamma^{\prime }\Sigma_{y}p\left(y|x,\pi \left(x\right)\right)V^{\pi }\left(y\right)$$

**Figure 4:**
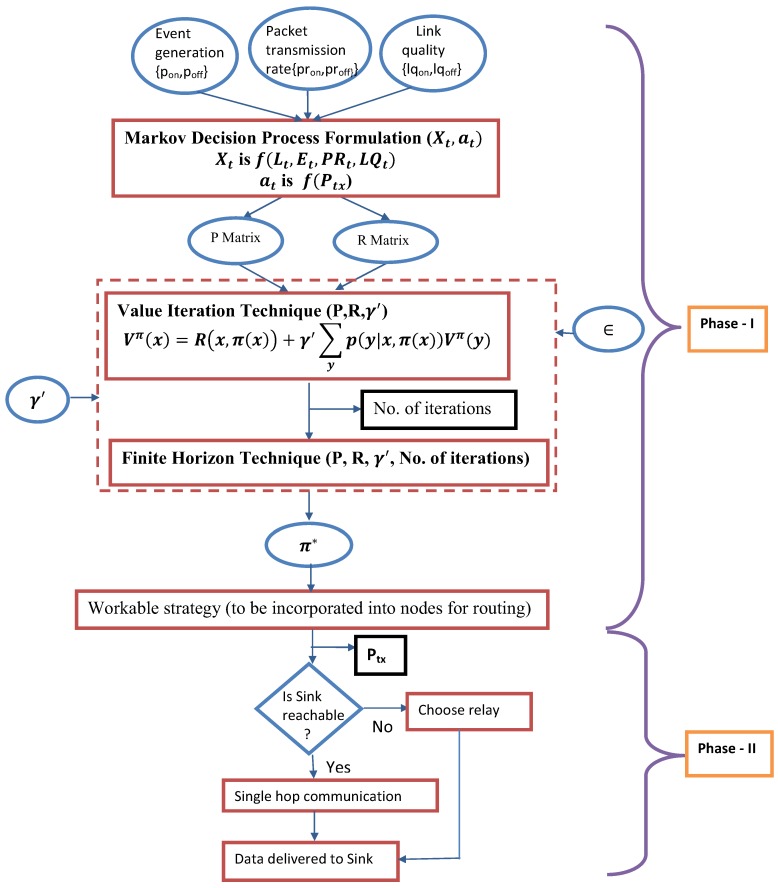
Work flow of MDP formulation of our work.

A few frequently used terms are listed in Table 2 along with their meaning for convenience. The steps followed are summarized in Phase I of Figure 4.

Initially, for a given combination of $p_{on}$, $p_{off}$, $pr_{on}$, $pr_{off}$, $lq_{on}$ and $lq_{off}$ the state transition table is constructed where all possible state transitions are recorded based on all possible actions ($a_{t}$) performed on all possible current states ($X_{t}$). Besides, rewards generated due to actions performed at each state are noted accordingly following Equation (9). At this point the process is provided with two matrices, i.e., state transition matrix (P) and reward matrix (R) which are then fed to value iteration technique. Value iteration function takes into account P, R and discount factor $\gamma^{\prime }$ as arguments and assigns an arbitrary value $V_{0}$ to each state which is repeated for all state *s*. In next iteration $V_{n+1}\left(s\right)$ is computed by Bellman backup at s [15] and the iterations are continued until $max_{s}\left|V_{n+1}\left(s\right)-V_{n}\left(s\right)\right|<\epsilon$ (i.e., ϵ convergence). This value iteration technique results in number of iterations and discounted utility values $U\left[\left(r_{0},r_{1},r_{2},\ldots \right)\right]=r_{0}+\gamma^{\prime }r_{1}+{\gamma^{\prime }}^{2}r_{2}+\ldots$. Herewith, finite horizon function is called which takes P, R, $\gamma^{\prime }$ and number of iterations as arguments and terminates after fixed number of steps and results in non-stationary policy (π) depending on time left. Finite horizon guarantees that for every policy, a terminal state will eventually be reached. The process can be repeated for different combinations of $p_{on}$, $p_{off}$, $pr_{on}$, $pr_{off}$, $lq_{on}$ and $lq_{off}$ to explore effective transmission strategies.

### 4.3. Phase II (Post-Deployment Phase)

In Phase II, the obtained policy of Phase I (denoted as $\pi^{*}$ in Figure 4) is incorporated into each node before deployment to initiate Phase II which focuses on developing network layer protocol. Thus, routing decisions could be made based on this pre-calculated policy to get long-term benefit in terms of energy consumption, successful data delivery and minimal thermal effect as well. This policy can be stored as a data structure where the optimal transmission power corresponding to each system state ($X_{t}$) would be listed. The optimal transmission strategy thus obtained can be fed to each node in WBAN as summarized in the routing protocol illustrated in Algorithm 1. The nodes when deployed, may tune themselves to the optimal transmission power according to their working conditions by simply looking into the data structure ($DS_{j}$). However, each node tries to establish connection with sink at the suggested optimal transmission power following MDP formulation for present system state. If it succeeds, data is transferred to the sink directly using the specified transmission power level. However, if sink is found to be not reachable at the suggested power level, it looks for relay nodes which are reachable with the same power level. $p_{on}$ and $p_{off}$ are mapped in Algorithm 1 using $flag_{on}$, $flag_{off}$. $flag_{on}$ is true to indicate data is sent in the previous time slot and data will be transmitted in the current slot with probability $p_{on}$; similarly $flag_{off}$ is true when there is no data transmission in previous slot and data will be transmitted in the next slot with probability $\left(1-p_{off}\right)$.

**Algorithm 1:** EstimateTransmissionPower ().
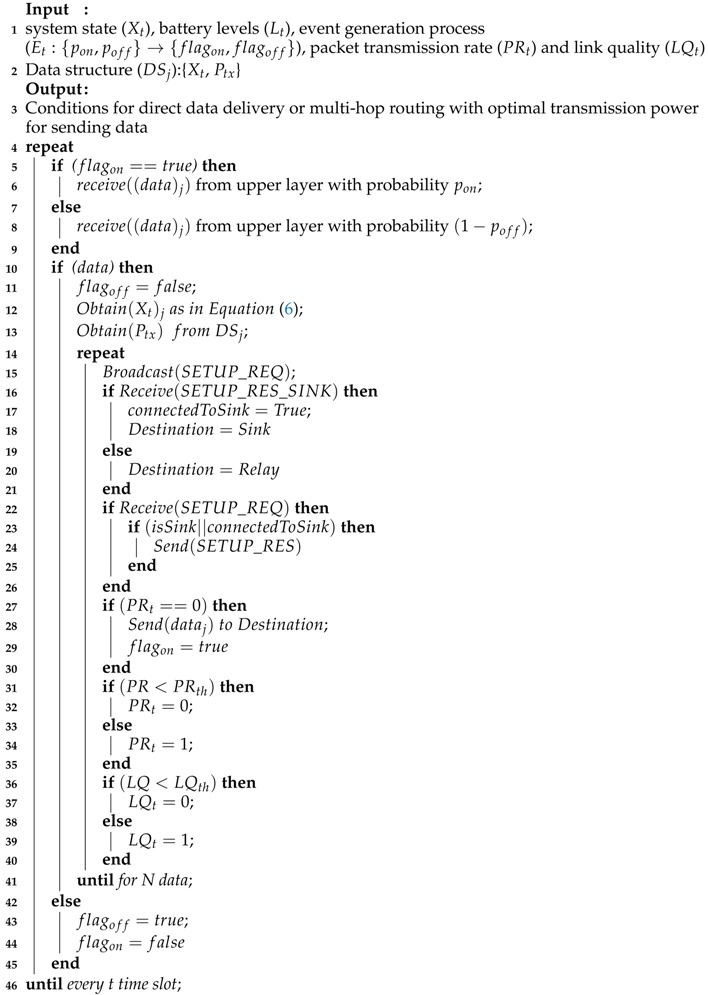


In the proposed algorithm, a node needs to execute simple local computations, conditional statements and simple table lookup that consume computation power denoted by $e_{compute}$, $e_{condition}$ and $e_{lookup}$ respectively. As noted in step 10 of Algorithm 1, if a node finds data to transmit in its queue, it will perform simple computational steps to come up with suitable transmission power (through steps 11–13) and broadcasts setup request if sink information is not cached. After receiving setup reply, the node can assign the sink or relay information and send data accordingly with given transmission power if it is not heated (according to step 28 of Algorithm 1) Thus, to send data, following the algorithm, $\left(2e_{condition}+4e_{compute}+e_{lookup}\right)$ is consumed initially and $\left(2e_{condition}+2e_{compute}+e_{lookup}\right)$ is consumed if the destination information is cached. In other words, to send a single data packet the associated computational complexity of the proposed algorithm is O (1). However, if n messages are sent in between two successive setup phases the computational complexity becomes O (n). Thus, the nodes need not perform complex computational steps such as fuzzy logic as in [17] or linear programming as in [33]. These works involve distance calculation between the nodes that require incorporation of path loss models. Moreover, generation of routing query, fuzzy inference system [17] or linear programming [33] are not simple conditional and assignment statements. Instead, each of these models requires multiple assignment and conditional statements, loops etc. that makes the system computationally complex. Thus, MDP formulation in the pre-deployment phase makes the routing algorithm for the nodes simple as far as the calculation of transmission power and decisions about single-hop and multi-hop are concerned.

## 5. Simulation Results

Accordingly, the process of implementation of the proposed approach is performed in two phases, i.e., implementing Phase I and then feeding the outcome of Phase I while simulating Phase II. Phase I includes the experiments related to the transmission strategy formulation mostly carried out using spreadsheets and R [34] software. In Phase II, a WBAN is simulated using Castalia 3.2 [35] simulator where each node is incorporated with the pre-computed strategy developed in Phase I. Nodes are programmed to follow Algorithm 1 to route data to the sink. Several experiments are performed in this phase to estimate the performance of the entire process in terms of the identified performance metrics.

### 5.1. Experimental Results of Phase I

Phase, I i.e., pre-deployment phase focuses on analytical formulation of transmission strategy with mathematical calculations before the probable states as defined in Equation (6) are put into effect. The experimental setup of Phase I includes MDP formulation with respect to the input conditions. At the beginning, the state transition matrix P containing all possible transitions from the current state ($X_{t}$) to the probable next states ($X_{t+1}$) depending on actions $a_{t}$ performed on $X_{t}$ have been formulated. Here four actions ($a_{t}\in \left\{0,1,2,3\right\}$) have been taken into account which are defined in terms of data packet transmission with transmission power −20 dBm, −15 dBm, −12 dBm, −10 dBm respectively (that are standards for WBAN defined by IEEE 802.15.4 [35]) which draw the remaining energy of a node into one of the corresponding five energy levels ($L_{t}\in \left\{0,1,2,3,4\right\}$). Hence, probability matrix (P) for each action is constructed as 40×40 matrix. The matrix includes all possible combinations of input variables considering five energy levels $L_{t}$, two probable values for event $E_{t}\in \left\{0,1\right\}$, packet transmission rate $PR_{t}\in \left\{0,1\right\}$ and link quality $LQ_{t}\in \left\{0,1\right\}$ respectively and thus 5×2×2×2 probable states could be defined. Next, reward matrix (R) is developed by estimating rewards following Equation (9) depending on actions $a_{t}$ performed at each system state $X_{t}$. In this phase the mathematical calculations are carried out first in Spreadsheet following Equations (1)–(14). The formulation thus formed is solved using value iteration and finite horizon methods, that are executed using R [34] software. R [34] is a simulation tool for statistical computing that has been exploited to carry out experiments of Phase I.

**Figure 5:**
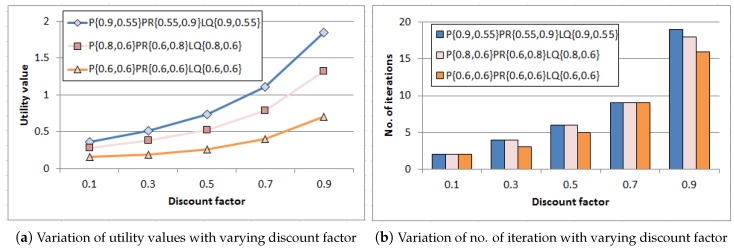
Performance of MDP with varying discount factor for different combinations of probability values related to event generation P (*p_on_*, *p_off_*), PR (*p_on_*, *p_off_*) and link quality LQ (*lq_on_*, *lq_off_*).

At this stage, a set of experiments have been done to study how discount factor ($\gamma^{\prime }$) puts an impact on discounted utility value with corresponding number of iterations. The value iteration process iterates until convergence to calculate the utility values for all states that got refined through approximation towards optimal value. Experiments have been performed for three representative input combinations of probability values corresponding to event generation process ($p_{on},p_{off}$), PR ($pr_{on},pr_{off}$), and link quality ($lq_{on},lq_{off}$). These depict the best-case, average-case and worst-case scenario (by regulating probability values) for event occurrence and successful data delivery to sink. Results are plotted in Figure 5a,b. As found in Figure 5a, utility value grows gradually for each combination with increasing discount factor $\gamma^{\prime }<1$ as sooner rewards have higher utility than later ones. In addition, smaller discount factor $\gamma^{\prime }<1$ leads to smaller horizon and hence the algorithm converges within few iterations which is reflected in Figure 5b as well. However, increasing discount factor $\gamma^{\prime }$ expands the horizon and the number of iterations intensifies accordingly for each combination which gives benefit in longer run.

**Figure 6:**
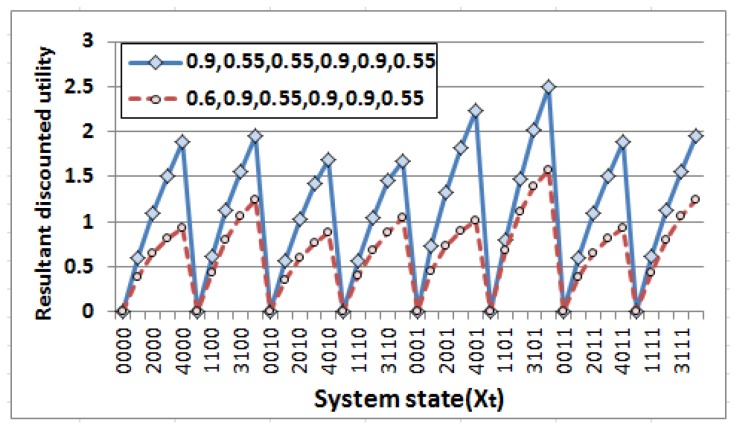
Variation of resultant discounted utility with system state for different combinations of probability values related to remaining battery power $L_{t}$, event generation P ($p_{on},p_{off}$), packet transmission rate ($pr_{on},pr_{off}$) and link quality ($lq_{on},lq_{off}$).

Another experiment has been carried out at this stage before deployment of the network to get insight about the resultant utility obtained from the value iteration process [15] corresponding to each state. The experiment has been performed for two different combinations of probability values ($p_{on},p_{off}$, $pr_{on},pr_{off}$, $lq_{on},lq_{off}$) as (0.9,0.55,0.55,0.9,0.9,0.55) and (0.6,0.9,0.55,0.9,0.9,0.55). The combinations are taken such that the values of ($p_{on},p_{off}$) pair denote high and low event generation probability respectively (to cover the entire horizon) whereas the other pairs, i.e., ($pr_{on},pr_{off}$) and ($lq_{on},lq_{off}$) remain unchanged at their best values to provide favorable conditions for data transmission. Results are presented in Figure 6. The system states represented as a combination of energy level $L_{t}\;\in \;\left\{0,1,2,3,4\right\}$, event generation $E_{t}\;\in \;\left\{0,1\right\}$, packet transmission rate $PR_{t}\;\in \;\left\{0,1\right\}$ and link quality $LQ_{t}\;\in \;\left\{0,1\right\}$ (as described in Equation (6)). Thus, system state (4,0,0,1) implies $L_{t}$ is 4, $E_{t}$ is 0, $PR_{t}$ is 0 and $LQ_{t}$ is 1. It is observed from the outcome that the resultant utility values corresponding to each state vary significantly for these combinations due to variation in the values of ($p_{on},p_{off}$) pair although other probabilities remain constant which indicates the impact of event generation probabilities in resultant discounted utility values. Furthermore, since the variation in energy level subject to the event generation probability and the action performed (illustrated in Equations (10) and (11)), the resultant discounted utility values are found to be zero when the system is at very low energy level, i.e., $L_{t}=0$. Hence, no reward is generated as there is insufficient energy to carry out any action (irrespective of events). Accordingly, the resultant utility values get larger corresponding to the system states with high energy levels and get maximized when $L_{t}=4$. In addition, similar pattern is observed for these representative combinations of probability values which mark two different horizon of event generation process. Hence, the obtained strategy, i.e., the optimal sequence of actions to be performed corresponding to each system state if incorporated into the nodes at the time of deployment, the system will work effectively. Measurable performance could be obtained irrespective of event generation probabilities when the other probabilities related to the PR and LQ are at favorable conditions.

Next, finite horizon function is performed in R [34] with formulated P and R matrix along with the number of iterations obtained from previous value iteration process as input to acquire optimal policy which indicates the end of pre-deployment phase as well.

### 5.2. Experimental Results of Phase II

**Figure 7:**
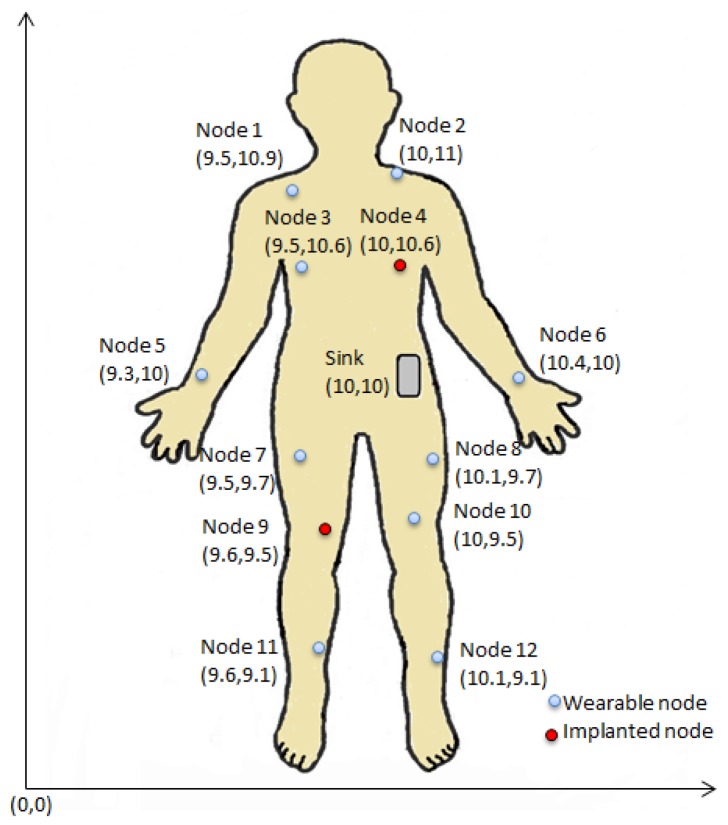
Node locations along with sink position.

The output obtained in Phase I is fed as input in Phase II, i.e., post-deployment phase where a WBAN configuration is simulated in Castalia 3.2 [35] which is a Wireless Sensor Network simulator based on OMNET++ platform useful for early phase algorithm/protocol testing. A total of 13 nodes including sink are deployed all over human body (around 10m×10m simulation area) out of which node 4 and node 9 are considered as implanted ones as shown in Figure 7 in such a way that they form a connected graph at the beginning where the sink node acts as BAN coordinator residing at roughly the center (i.e., waist). The network size follows the typical medical network based on WBAN which consists of 6 to 20 nodes (maximum) [2]. The default parameters used in the experiments are listed in Table 3 and any alterations to these values are stated explicitly. Mobility of each node due to posture change is modeled with LineMobility model [35]. The effective transmission power for each data communication is obtained from the set of transmission power levels {−20dbm,−12dbm,−15dbm,−10dbm} defined by BANRadio (i.e., the radio module of Castalia 3.2 [35] for WBAN communication) to be operated with IEEE 802.15.4 ZigBeeMAC protocol. Accordingly, the transmission range corresponding to each power level is governed by BANRadio [35] as well. Although the experimentations are carried out based on IEEE 802.15.4 ZigBeeMAC but the proposed approach can work with other existing technologies (for instance Bluetooth Low Energy (BLE) [2]). The SAR is quantified for each node following Equations (4) and (5). The optimal set of actions depending on $X_{t}$ of a node as obtained in Phase I is incorporated in each node during deployment following Algorithm 1 to minimize computational complexity and maximize lifetime without degrading the performance.

**Figure 8:**
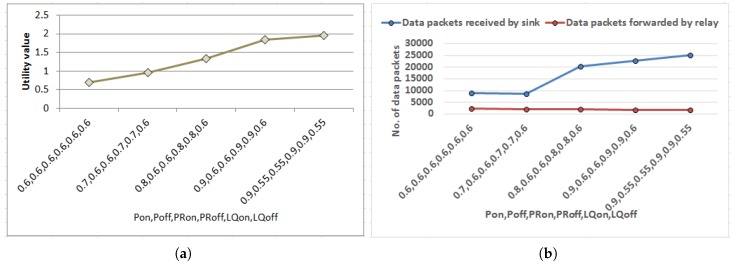
Mapping of MDP formulation into routing strategy. (**a**) Variation of utility values with varying $p_{on},p_{off},pr_{on},pr_{off},lq_{on},lq_{off}$. (**b**) Variation of data packets received by sink with varying $p_{on}$, $p_{off}$, $pr_{on}$, $pr_{off}$, $lq_{on}$, $lq_{off}$.

Herewith, a series of experiments have been carried out to validate the proposed strategy as well as to estimate performance with respect to the state-of-the-art work. Data packets received by sink has been chosen as a metric to quantify the performance of the proposed approach. At the beginning, experiments have been done to determine ideal combinations of the probability values, i.e., $p_{on},p_{off},pr_{on},pr_{off},lq_{on},lq_{off}$ in relation to event generation, packet sending rate and LQ respectively as illustrated beforehand. As depicted in Figure 8a utility values corresponding to each combination are presented in ascending order. The corresponding performance of each combination when mapped into routing strategy measured in terms of data packets received by sink has been plotted in Figure 8b. Other metric data packets forwarded by relay nodes are also included in Figure 8b to study the behavior of the multi-hop routing. Results show that performance of the proposed strategy when simulated (in Figure 8b) enhanced following similar pattern as predicted before deployment of the network presented in terms of utility values in Figure 8a. This justifies the correctness of mapping of the mathematical formulations into routing approach. In addition, when data packets forwarded by relay nodes are observed corresponding to implementation of each probability combination, marginally more forwarding traffic is found in adverse situations as regulated by the probability values to sustain the performance of the proposed strategy. Accordingly, the combination {0.9,0.55} for $\left\{p_{on},p_{off}\right\}$, {0.55,0.9} for $\left\{pr_{on},pr_{off}\right\}$ and {0.9,0.55} for $\left\{lq_{on},lq_{off}\right\}$ having highest utility value and corresponding maximum data delivered to sink have been selected as reference values to carry out the subsequent experiments. For instance, the reference value for $p_{on}$, i.e., 0.9 is implemented in the experimental setup using $flag_{on}$ and a random number generator which generates any number between 0 to 9. When there is data in the previous time slot $flag_{on}$ is set as true and the probability of data generation in the next slot is regulated with probability of occurrence of any number from 0 to 9 using random number generator except any particular number such as 5.

**Figure 9:**
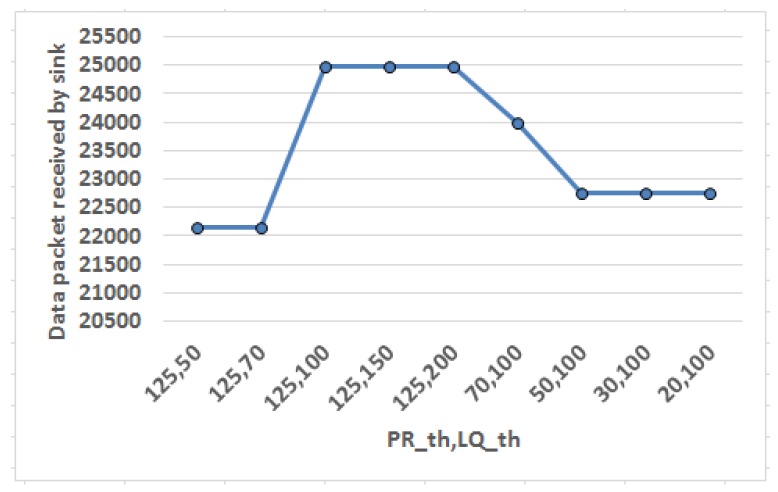
Obtaining threshold values for packet transmission rate ($PR_{th}$) and link quality ($LQ_{th}$).

Next experiment attempts to fix threshold values for packet transmission rate ($PR_{th}$) and link quality ($LQ_{th}$) as shown in Figure 9. Here X axis represents the threshold value pair ($\left\{PR_{th},LQ_{th}\right\}$) and the effect of variation in these threshold value pair in system performance is quantified in terms of data packets received by sink. Threshold value is a positive value set as reference point such that the obtained values can be compared with respect to the threshold to determine whether it violates its regulatory limit. Packet transmission rate (PR) is measured in terms of number of packets (having size of 2000 bits each) sent per second. Initially, $PR_{th}$ is set as a default large value, i.e., 125 packets/s (in other words 250 kbps which is maximum permissible data rate for IEEE 802.15.4 standard [2]) to adjust $LQ_{th}$ first. Link quality (LQ) is quantified in terms of link quality indicator (LQI) which is a metric to measure quality of the received signal. Gradually the $LQ_{th}$ is varied keeping $PR_{th}$ fixed at 125 and the effect is observed. It is found that data packets received by sink saturates when $LQ_{th}$ is beyond 100 and hence it is set as reference for $LQ_{th}$. Thereafter, $PR_{th}$ is varied in descending order keeping $LQ_{th}$ constant at reference value and the behavior is noticed. It is found that data packet reception by sink grows sharply when $PR_{th}$ is beyond 50. Hence, the reference value for $PR_{th}$ is set as 50 to bound PR to restrict SAR.

**Figure 10:**
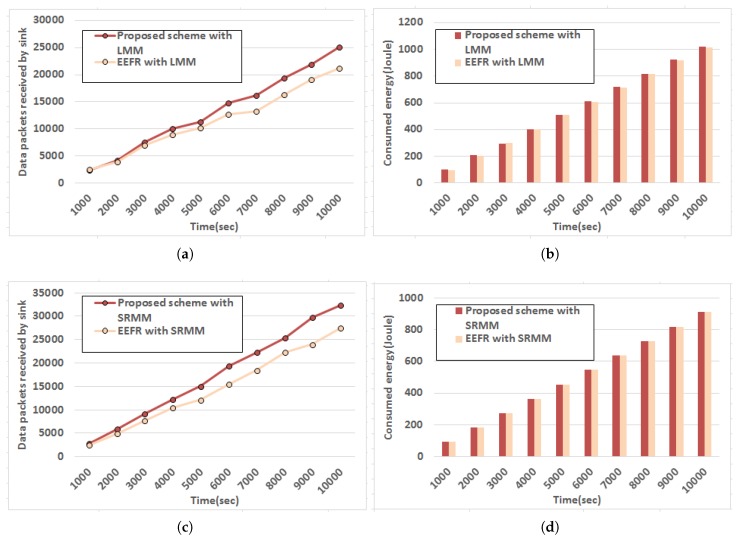
Performance evaluation of the proposed routing strategy with respect to time. (**a**) Variation of data packets received by sink with time following Line Mobility Model (LMM). (**b**) Variation of energy consumption with varying time following LMM. (**c**) Variation of data packets received by sink with time following Smooth Random Mobility Model (SRMM). (**d**) Variation of energy consumption with varying time following SRMM.

Following set of experiments are carried out to estimate the performance of the proposed strategy subject to different mobility models defined by Line Mobility Model (LMM) [35] and Smooth Random Mobility Model [36]. The nature of data packets received by sink with respect to time is observed here in case of LMM and Smooth Random Mobility Model (SRMM) and the outcome is plotted in Figure 10a,c respectively and corresponding energy consumption of the network measured using resourseManager module in Castalia [35] (that models realistic node behavior to access the radio) is depicted in Figure 10b,d accordingly. A recent protocol presented in [17] is also simulated in similar experimental setup to compare performance. It is observed from the outcome that relatively better performance in terms of data packets received by sink is achieved by both approaches while SRMM is followed with marginally less energy expenditure since in SRMM movement pattern does not have any sharp turn or sudden stop. Besides, the speeds are also increased gradually. Thus, LQ is not changed drastically when SRMM is followed to model body movement due to posture change. However, the proposed approach exhibits better performance in terms of data packets received by sink than the existing protocol [17] though the amount of energy consumption is comparable.

In the subsequent experiment, the proposed strategy is evaluated in terms of heat generation due to network activities. For this experiment, another metric heating ratio has been introduced which is defined as follows

(16) $$Heating\;ratio_{i}\;=\frac{\sum_{SAR_{i}>SAR_{th}:i\in n}Time_{i}}{\sum Time}$$

**Figure 11:**
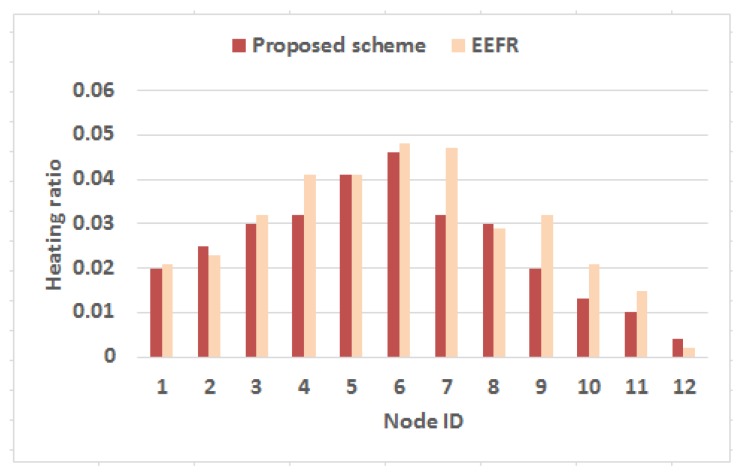
Heating ratio of each node in the network.

Heating ratio ($Heating\;ratio_{i}$) of any node *i* in the network of *n* nodes is evaluated as the summation of the discrete timespans for which the SAR results due to node *i* ($SAR_{i}$) measured following Equations (4) and (5) exceeds its regulatory limit (given in Table 3) with respect to the entire simulation period. The outcome is plotted in Figure 11. It is found from the outcome that the nodes in the network exhibit much low heating ratio as they got heated for small duration while following the proposed strategy. However, the nodes which are closer to sink as shown in Figure 7 for instance node 6, node 7, node 4, etc. produce relatively high heating ratio with respect to other nodes in the network as they often work as forwarder for others as well exploiting their connectivity to sink. Even, the proposed approach shows low heating ratio as compared to the state-of-the-art energy-efficient protocol [17]. Hence, the objective to prevent thermal damage of human tissue is achieved with the designed multi-hop transmission strategy.

Finally, the behavior of the proposed strategy is observed with growing network size. Reliability of the proposed work is quantified here in terms of Packet Delivery Ratio (PDR) which is defined as the ratio between total number of packets received by sink to the sum of the packets sent by the nodes presented as follows

(17) $$PDR\;=\;\frac{\sum_{s}data_{received}}{\sum_{i=1}^{n}data_{sent}}$$

**Figure 12:**
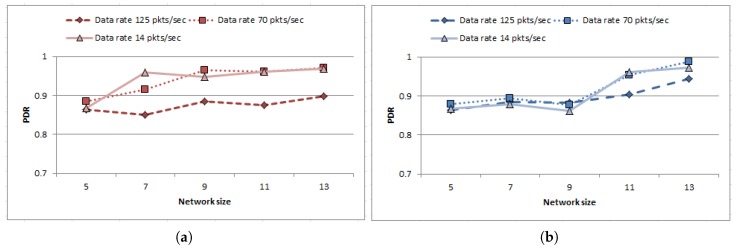
Reliability assessment of the proposed routing strategy in terms of Packet Delivery Ratio (PDR) with respect to growing network size. (**a**) Variation of PDR with growing network size following LMM. (**b**) Variation of PDR with growing network size following SRMM.

The experiments started with 5 nodes having connected topology at the beginning and gradually more nodes are included to form a bigger network (up to default network size) to estimate the scalability of the proposed approach. The experiments were carried out following LMM [35] and SRMM [36] for three different data sending rates 14 packets/s, 70 packets/s and 125 packets/s having packet size 2000 bits each (i.e., in other words 28 kbps, 140 kbps and 250 kbps). Results are plotted in Figure 12a,b. Since the number of relay nodes increases with growing network size which enhances the chances to find route to destination for the data traffic, the performance of the proposed protocol improves irrespective of the data sending rate in case of both mobility models. Hence, it is evident from the outcome that the proposed strategy can cope up with the increasing network size while ensuring substantial reliability in terms of PDR.

Herewith, the entire experimental procedure can be perceived as an integration of two phases namely Phase I and Phase II. In Phase I, transmission strategy for intra-BAN communication has been formulated following MDP and experiments are conducted to analyze the behavior of MDP parameters (discount factor ($\gamma^{\prime }$), number of iteration) and output (utility value calculated for several iterations given a discount factor) subject to varying input conditions. In Phase II, the proposed approach is simulated. Here the strategy obtained from Phase I (in terms of optimal transmission power corresponding to each system state $X_{t}$) has been incorporated to the nodes before deployment of the network.

Theoretical analysis of Phase I has been mapped to the simulated outcome of Phase II for the first two experiments of Phase II shown in Figure 8 and Figure 9. The results in Figure 8 validate the prediction by MDP when the nodes are actually routing data to yield maximum utility (mapped to data packets received). Next experiment finds out the tunable values of threshold parameters as shown in Figure 9.

Subsequent experiments are conducted to find out the effectiveness of the transmission strategies with respect to routing data. Performance metrics such as data packets received by sink, consumed energy, heating ratio, and reliability in terms of PDR are observed with respect to time and increase in network size subject to relative node movement. Values of the tunable parameters for the overall experimental process are listed in Table 4.

## 6. Conclusions

Widespread deployment of IoT in medical applications requires effective handling of challenges related to planning, developing, and managing solutions for medical IoT. Strategies are to be developed for communications among energy-constrained body-sensor nodes (or things) within WBAN for making medical IoT green (or energy efficient) without degrading the performance. In this paper, an energy-efficient transmission strategy has been formulated following MDP which effectively determines the actions to be performed described in terms of acquiring optimal transmission power for intra-BAN communication based on the system state defined as quadruplets of current energy level of the node, event occurrence, PR of the node and LQ. The policy is designed offline using value iteration process and incorporated into the nodes to be reflected at the time of making routing decisions. This strategy enables planning under uncertainty with minimum computational overhead. The proposed approach predicts the favorable conditions for multi-hop routing over single-hop direct data delivery to achieve long-term benefits in energy consumption. The algorithm is validated through extensive simulations and the proposed approach is found to outperform the state-of-the-art work in terms of data packets received by nodes. In addition, the proposed routing approach can restrict heat generation as well. The future work plan will include more dimensions in strategy formulation.

## Figures and Tables

**Table 1:** Research work on developing transmission strategies published since 2009.

Year	Existing Work	Network	Topology Used	Issues Handled	Mathematical Model Used	Input Conditions	Performance Metric
2009	Generic model [11]	WSN	Single-hop	Energy replenishment	Markov model	Energy status	Battery capacity, reward rate
2010	Transmission strategies [12]	WBAN	Single-hop	Energy harvesting, energy efficiency, reliability	Markov model	Current energy level, state of data generation process, battery recharge, packet error probability	Quality of coverage
2011	Selective forwarding [22]	WSN	Multi-hop	Energy effiviency	Markov Decision Process	available battery of the node, the energy cost of retransmitting a message or the importance of messages	Suboptimal scheme and reduced computational cost
2012	Transmission policies [13]	WSN	Single-hop	Energy harvesting	Markov model	Current energy level of sensors, data importance	Transmission probability, energy level
	Routing protocol [18]	WBAN	Multi-hop	Energy efficiency, power control, transmission reliability, low overhead	CTP	Changing link quality, end to end delay, packet loss	Packet reception ratio, delay, energy consumption, energy balancing
2013	Transmission policies [14]	WSN	Single-hop	Energy harvesting	Markov model	battery capacity, data transmission with a given energy cost	Asymptotic average reward as a function of SNR, transmission probability
	Routing protocol [17]	WBAN	Multi-hop	Energy efficiency, power control, lifetime	-	Distance of the receiver	Remaining energy
2015	Transmission policies [24]	WSN		Energy harvesting	Markov model	Energy level, data queue	Buffer size and battery, large data buffer case, low complexity policy
	Transmission approach [27]	WBAN	Single-hop	Energy efficiency		Circuit energy, transmission energy on distance	Energy consumption, recovery energy, transmission time, duty cycle
2016	Transmission policies [26]	WSN	Multi-hop	Energy efficiency	Network coding	No. of relay, recoding scheme and field size, Source and relay transmission	Mean transmission, medium access probability
2017	Transmission strategies [28]	WBAN		Energy efficiency	Discrete Markov Arrival Process	Channel state, battery state, no. of buffered packet in the system	
	Optimizing transmission [7]	WBAN	Multi-hop	Transmission reliability, energy efficiency, lifetime, body movement		Signal to noise ratio, bit error rate	Transmission success rate, packet size, sensed data percent, burden packets per sec, transmission reliability, energy efficiency, energy consuming speed, energy balance degree, lifetime
2018	This study	WBAN	Multi-hop	Transmission power, energy efficiency, body movement, heat generation	Markov Decision Process	Energy level, event generation, packet transmission rate, link quality	Packet received by sink, consumed energy, packet delivery ratio, heating ratio

**Table 2:** Description of frequently used terms.

Terms	Description
$X_{t}$	A finite set of states
$A_{t}$	A finite set of actions $\left(a_{t}\right)$ to be taken
*P*	Transition probability matrix, where the state transitions are given by $p\left(x_{\left(t+1\right)}|x_{t},a_{t}\right)=p\left(x_{\left(t+1\right)}|x_{0}\ldots x_{t},a_{0}\ldots a_{t}\right)$. This matrix plays the key role in finding the next state $x_{\left(t+1\right)}$ which is considered to be a possible consequence of performing an action $\left(a_{t}\right)$ in a state $\left(x_{t}\right)$. Hence it is depicted as a set of square matrices one for each action having both dimensions indexed by states.
*R*	Reward matrix where each entry gives the immediate reward (or expected immediate reward) $r(x_{t},a_{t})$ received for state transition from $x_{t}$ to $x_{\left(t+1\right)}$ performing action $a_{t}$.
$\gamma^{\prime }$	[0,1] Discount factor denoting the importance of future reward in present reward.
Π(x)	A policy Π gives an action for each state x, $\Pi^{*}\left(x\right)$ is optimal policy, i.e., the sequence of actions which maximizes expected utility if followed
$U_{dis}\left(x\right)$	Expected discounted resultant utility value at each state obtained using value iteration process
$P_{tx}$	Transmission power
$p_{on}$	[0.5,1]Probability of occurrence of an event in next slot when there is an event in present slot
$p_{off}$	[0.5,1]Probability of occurrence of no event in next slot when there is no event in present slot
$pr_{on}$	[0.5,1]Probability of exceeding maximum limit of packet transmission rate, $PR_{th}$ in next slot when $PR_{th}$ exceeded in current slot
$pr_{off}$	[0.5,1]Probability of not exceeding $PR_{th}$ in next slot when $PR_{th}$ not exceeded in current slot
$lq_{on}$	[0.5,1]Probability that indicates stable channel condition in next slot when link quality is above threshold ($LQ\;>\;LQ_{th}$) in present slot
$lq_{off}$	[0.5,1]Probability that indicates unstable channel condition in next slot when link quality is below threshold ($LQ\;<\;LQ_{th}$) in present slot
$E_{rem}$	Remaining energy of a node

**Table 3:** Simulation parameters and their default values.

Simulation Parameter	Default Value
Simulation area	10m×10m
Simulation time	10,000 s
Network size	13
Mobility model	LineMobility model [35]
MAC protocol	IEEE 802.15.4
Data generation rate	14 packets/s
$SAR_{th}$	0.3357 Watt/Kg [3]

**Table 4:** Tunable parameters and their values.

Tunable Parameters	Tunable Values
**Phase I:**
$\gamma^{\prime }$	0.9
number of iterations	Up to 19
**Phase II:**
PR	10 kbps to 125 kbps
$PR_{th}$	50
$LQ_{th}$	100
